# Barriers and facilitators to the use of personal information documents in health and social care settings for people living with dementia: A thematic synthesis and mapping to the COM‐B framework

**DOI:** 10.1111/hex.13497

**Published:** 2022-04-12

**Authors:** Emily Clark, Fiona Wood, Suzanne Wood

**Affiliations:** ^1^ Division of Population Medicine, School of Medicine Cardiff University Cardiff UK; ^2^ Cardiff and Vale Local Public Health Team Public Health Wales Cardiff UK

**Keywords:** communication, continuity of patient care, dementia, health records personal, patient‐centred care, personal information documents, systematic review

## Abstract

**Introduction:**

People living with dementia experience communication difficulties. Personal information documents, or healthcare passports, enable communication of information essential for the care of a person with dementia. Despite the potential for providing person‐centred care, personal information documents are not ubiquitously used. The Capability Opportunity Motivation—Behaviour (COM‐B) model can be used to understand factors determining individuals' behaviours.

**Objectives:**

This study aimed to identify the barriers to and facilitators of the use of healthcare passports for people living with dementia through a systematic review methodology.

**Methods:**

A systematic search of six electronic databases was undertaken. Grey literature was searched using three databases. All study types reporting barriers to or facilitators of the use of personal information documents in the care of adults living with dementia in high‐income countries were included. Study quality was assessed using the NICE Quality Appraisal Checklist. Thematic synthesis was used to develop descriptive themes, which were subsequently mapped to the COM‐B framework.

**Results:**

Nineteen papers were included. Themes included training, awareness, embedding the process in norms and appreciating the value of the personal information documents. A broad range of barriers and facilitators was identified within each COM‐B domain.

**Conclusion:**

This framework provides a starting point for evidence‐informed initiatives to improve the use of personal information documents in the care of people with dementia.

**Patient and Public Contribution:**

This is a review of studies and did not involve patients or the public. Review results will guide evaluation of a local personal information document, which will be designed with input from the Dementia Champions Network (includes carers and other stakeholders).

## 1 INTRODUCTION

Dementia is a public health priority according to the World Health Organization.^1^ With an ageing population, improved levels of awareness, and earlier diagnosis, the prevalence is anticipated to increase.^2^ Characterized by cognitive impairment, changes to behaviour and personality, communication difficulties and problems with activities of daily living,^2^ there is an associated need for high‐quality specialist care.

Between 25% and 50% of hospital inpatients at any given time in England are people with a diagnosis of dementia.^3^, ^4^ People living with dementia (PLWD) experience higher rates of morbidity and mortality during inpatient admissions.^2^, ^5^ Hospital admission places the individual in unfamiliar surroundings, which can be frightening and disorientating, removes PLWD from their routines and habits and separates individuals from their families and carers.^3^ This is particularly true during the severe acute respiratory syndrome coronavirus 2 (SARS‐CoV‐2) pandemic, where carers and families experienced difficulties visiting PLWD at home and in hospital.^6^

Variation in needs between PLWD due to differing symptoms and stages of the disease and variation in support available to the individual compound the difficulty in providing person‐centred care and highlight its importance. The ‘person‐centred approach’ was first coined by Kitwood and forms a mainstay of high‐quality care for PLWD.^7^ The VIPS framework^8^ describes person‐centred care for PLWD, and underpins NICE recommendations.^2^ VIPS involves: ‘**V**aluing people with dementia and those who care for them; treating people as **I**ndividuals; looking at the world from the **P**erspective of the person with dementia; a positive **S**ocial environment in which the person living with dementia can experience relative wellbeing’.^2^

PLWD may be unable to communicate their needs and preferences themselves. The Royal College of Psychiatrists advise use of tools to capture essential information about a PLWD.^9^ Such documents are often called ‘personal information documents’ or ‘healthcare passports’, amongst other terms.^9^, ^10^ Personal information documents (PIDs) take various forms, such as booklets or leaflets. They are designed to be completed by the PLWD, family or carers, or healthcare professionals (HCPs) involved in their care. Information such as preferred name, likes and dislikes, assistance needed with activities of daily living and routines is captured.^9^ This enables essential information to be swiftly transferred between settings, such as between the home and the hospital. For example, food preferences can be easily communicated, thereby reducing complications arising from poor eating and drinking.^3^

The 2018–2019 audit by the Royal College of Psychiatrists in England and Wales observed suboptimal collection of essential personal information, with only 48% noting food and drink preferences, 36% documenting triggers for distress and 32% reporting methods to calm and reassure the PLWD.^11^ A Care Quality Commission report found that use of PID was varied, even within hospitals.^4^

Effective use of a PID requires behavioural changes amongst several stakeholders (patients, carers and HCPs). The Capability, Opportunity, Motivation—Behaviour (COM‐B) model is a theory of behaviour that contributes insights into the challenges of using a PID in a variety of contexts.^12^ Positioned at the heart of the behaviour change wheel, the COM‐B model is used for intervention development;^12^ for example, the COM‐B model has been previously successfully used in improving dementia care in hospitals.^13^ The model identifies interrelationships. For example, staff training (physical capability) influences awareness and understanding of PID (psychological capability), and may influence HCP attitude (automatic motivation), HCP experience of PID (reflective motivation) and organizational culture (social opportunity).

Through a systematic review methodology and thematic synthesis, this review aims to address the research question: what are the factors that create barriers and facilitators in specific contexts to the use of healthcare passports for PLWD? We then aimed to map those factors onto the COM‐B model.

## 2 MATERIALS AND METHODS

The review protocol was prospectively registered with PROSPERO (Registration number: CRD42020193287; URL: https://www.crd.york.ac.uk/prospero/display_record.php?ID=CRD42020193287). The review was conducted and reported in accordance with ENTERQ guidelines^14^ (see Table S1 ENTREQ checklist). We used principles of thematic synthesis^15^ to bring together studies that report factors relating to the barriers and facilitators of the use of PIDs from the perspective of a range of stakeholders (PLWD, carers and HCPs).

### 2.1 Approach to searching

Searches were preplanned, with a comprehensive search strategy developed to identify all available studies. Therefore, this review was designed to promote inclusivity.

### 2.2 Electronic search strategy

A scoping search identified that the lack of a unifying term for PID was problematic. With help from a specialist librarian, a comprehensive and sensitive search strategy was developed. A copy of the search strategy for OVID Medline can be found in Table S2. Key words and Medical Subject Headings terms for the three main topic areas (adults living with dementia or cognitive impairment, their carers or health professionals; PIDs; qualitative research or mixed‐methods research) were combined, according to the inclusion criteria.

### 2.3 Inclusion criteria

Inclusion and exclusion criteria were determined *a priori*. The population of interest included adults living with dementia or cognitive impairment, their carers and HCPs. The term ‘carers’ is used throughout the review to refer to adult informal and unpaid carers, usually family or friends of the PLWD.^1^ We included studies conducted in high‐income settings (according to the World Bank's income classification) and included community, care home, primary care and hospital.

The intervention of interest was a PID, defined for the purposes of this review as a document assisting essential care through the communication of key information about the PLWD (such as their preferred name), their preferences and routines. Documents with a primary aim of communication of clinical information between HCPs or focussing on end‐of‐life care or advance directives were excluded. PIDs included within a toolkit of interventions (referred to here as multicomponent studies) were included for completeness. These studies were analysed separately in parallel to single‐component intervention studies as the barriers/facilitators may differ due to the presence of additional interventions and the difficulty in extracting data specific to the PID as opposed to other intervention components.

Qualitative, quantitative and mixed‐methods studies reporting barriers and facilitators were included. We included published studies and reports, and excluded those that had only been published as conference abstracts or unpublished PhD or Masters theses. Studies had to be published in English (the language spoken by the review team) to ensure that themes were appropriately identified, understood and represented. Date limits were applied from 2010 to the present, as the Royal College of Psychiatrists recommended PID use following their 2010–2011 National Audit of Dementia.^9^ In addition, the Alzheimer's Society developed and launched ‘This is me’, their PID in 2010,^16^ and this was endorsed by the Royal College of Nursing in 2010.

### 2.4 Data sources

The following databases were searched: CINAHL, HMIC, MEDLINE, PsycINFO, Scopus, Web of Science to cover a range of healthcare, social science and nursing journals. Grey literature databases EThOS, OpenGrey and GoogleScholar were also searched as we felt that relevant data might be available in websites and project reports of dementia‐related charities. Reference lists of relevant studies were hand searched for further articles or resources that might have been pertinent. In three cases, no full text accompanied relevant abstracts and so authors were contacted. Two responded, but full‐text articles did not fulfil the eligibility criteria. Databases were searched between 2 July 2020 and 9 July 2020, and an updated search was conducted between 7 March 2022 and 9 March 2022. OVID was searched in 2022, in place of searching Medline, PsycInfo and HMIC separately.

### 2.5 Study screening

Records identified from database and website searching were imported into EndNote Online. Records were deduplicated and titles clearly irrelevant to the research question were excluded by the lead author. The lead author reviewed abstracts and full texts against eligibility criteria. The reasons for exclusion were documented. A second reviewer independently reviewed 10% of abstracts and full texts. Disagreements were discussed. Records eligible at abstract review were reviewed at full text using the same process.

### 2.6 Data extraction

Data from the results sections of primary studies were extracted. We used a data extraction table, which included quality score, research question, study methodology, results (including themes identified) and limitations.^17^ Data extraction tables were completed by the lead review author; 10% were independently checked by the second author for consistency.

### 2.7 Quality appraisal

Studies were quality appraised using the checklist from ‘Methods for the development of NICE public health guidance’^17^ to assess their robustness of their conduct. The lead author appraised each paper for quality, and two papers were independently assessed by the second author for consistency. Each paper was then assigned an overall assessment score (++, +, −). There are no validated mechanisms to select studies based on their quality score for qualitative reviews,^15^ and so all studies were included irrespective of score. In addition, the priority for the review was generating breadth of opinion including discordant observations.

### 2.8 Analysis and synthesis

A thematic synthesis was conducted.^15^ This method draws on concepts used for thematic analysis in qualitative research in primary studies as a method of identifying and developing themes within the data. This is an accepted method to review qualitative studies as the links between the primary studies and final synthesis are maintained.^18^ Analyses were conducted separately for papers describing single‐ and multicomponent interventions. First, data from the results sections of papers were inductively coded using a line‐by‐line method according to the meaning and content by the lead author, supported by qualitative data software NVIVO v12. Direct quotes were all coded, as were author statements.^19^ Codes were structured (in tree form) or in free form (without hierarchical structure). New codes were created as necessary as we progressed through the papers and similar, or related, codes were grouped. If research was reported in a journal article as well as a research report, both were included. A second reviewer read and coded two of the papers. Although the initial analysis was conducted by the lead author, the analysis was reviewed in discussion with all review authors. The descriptive inductive themes were further developed by grouping codes with similar meanings. As barriers and facilitators often described the same phenomenon from different angles, they were analysed together; however, it was noted whether the quote illustrated a barrier or enabler.

Descriptive themes were then mapped onto the COM‐B model for behaviour change^12^ to develop an analytical framework for the barriers and facilitators to healthcare passport use, from the perspectives of PLWD, carers and HCPs. The COM‐B model is used within the behaviour change wheel, which was designed specifically to support behaviour change interventions.^12^

## 3 RESULTS

**Figure 1:**
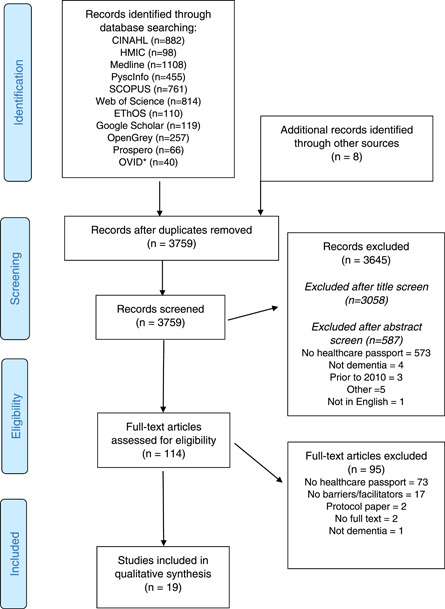
Preferred Reporting Items for Systematic Reviews and Meta‐Analyses flow diagram. Adapted from Moher et al.^21^ and searched in 2022 only

The original search, conducted in July 2020, identified 3924 records (2971 after deduplication) The search was updated in March 2022 and a further 789 papers were included for screening. Eighteen studies were included in qualitative synthesis following the original search, and one additional study^20^ was identified following the updated search in 2022. Consequently, 19 papers were included in the final review (see Figure 1), of which 11 detailed a single‐component intervention and 8 described a PID as part of a multicomponent intervention. Table 1 presents a summary of the included single‐component studies; Table 2 summarizes multicomponent studies.

**Table 1:** Summary of single‐component studies

Reference quality	Country	Setting	Perspective	Tool	Participants	Design		Theoretical models	Analysis
Baillie and Thomas (2020)^22^ QS: +	England (NHS)	Hospital/community	HCP	This is me (from Alzheimer's Society)	Nurses, AHPs, doctors and nonclinical staff in 12 focus groups (*n* = 58); 1 interview	Focus group/interviews following dementia awareness training		Social constructionist	Secondary thematic analysis
Bray et al. (2015)^23^ QS: −	England (NHS)	Hospital	HCP	Patient passport (adapted by NHS Trust)	Hospital staff	Case study		None stated	None stated
Brooker et al. (2014)^24^ QS: +	England (NHS)	Hospital	HCP	Patient passport	Nurses	Case study within evaluation of dementia care		None stated	None stated
Burton et al. (2010)^25^ QS: +	Australia	Hospital	HCP	I AM form (renamed: Focus on the person form)	Hospital staff from medical wards	3x focus group		None stated	Thematic analysis
		Community	Carers		Carers responding to adverts in carer networks	Interview at 1 and 4 weeks		None stated	Thematic analysis
Clinical Excellence Commission^a^ (2014)^26^ (research report) QS: +	Australia	Hospital	HCP	TOP 5 (developed by the study team)	Clinicians (at least 6 per survey) at 17 public hospitals; 4 private hospitals	Quantitative and qualitative (free text in survey) data collected at baseline, 6 and 12 months. LSL midway and process reports	None stated		Thematic analysis of free‐text survey responses
			Carers		Carers invited by staff during admission	Carer survey during acute care admission	None stated		As above
Clinical Excellence Commission (2015)^27^ QS: +	Australia	Hospital	Carers	TOP 5 (developed by the study team)	Carers of PLWD during admission	Survey during admission		None stated	Thematic analysis of free‐text survey responses
		Hospital	HCP		Clinicians including preadmission, perioperative, ED staff	Survey at baseline, 6 and 12 months. LSL midway and final reports		None stated	As above
		Care home	HCP		Staff at care home	As above		None stated	As above
		Community	HCP		Community staff	As above		None stated	As above
		Ambulance service	HCP		Paramedics	As above		None stated	As above
Leavey et al.^c^ (2017)^10^ (research report) QS: +	Northern Ireland (NHS)	Community	PLWD and carer	Healthcare passport (developed by the study team)	Purposive sample of PLWD and carers; 26 patient–carer dyads	Longitudinal qualitative research and 3x in‐depth interviews at baseline, 6 and 12 months		Realist evaluation approach to complex interventions	Trajectory analysis of interviews; informed by prior realist review using the framework approach. Document content analysis of entries in HP
		Primary care	HCP		GPs of PLWD/carer dyads; *n* = 5	Interviews with GP of PLWD at the end of the study, that is, 18 months		As above	Trajectory analysis
Leavey et al.^d^ (2020)^28^ (research article) QS: +	Northern Ireland (NHS)	Community	PLWD and carer	Healthcare passport developed by study authors	Purposive sample of PLWD and carers; 26 patient–carer dyads	Longitudinal qualitative research and 3x in‐depth interviews at baseline, 6 and 12 months		Realist evaluation approach to complex interventions	Trajectory analysis of interviews. Document content analysis of entries in HP
		Primary care	HCP		Not stated	Interviews with GP of PLWD at the end of the study, that is, 18 months		As above	Trajectory analysis
Luxford et al.^b^ (2015)^29^ (research article) QS: +	Australia	Hospital	HCP	TOP 5 (developed by the study team)	Clinicians (at least 6 per survey) at 17 public hospitals; 4 private hospitals	Survey at baseline, 6 and 12 months		None stated	Thematic analysis
			Carers		Carers invited by staff during admission	Survey during admission		None stated	As above
McGilton et al. (2017)^30^ QS:+	Canada	Care home	HCP	Resident‐centred communication intervention developed by the study team	HCP in long‐term care home	Focus group/interviews		Action research implementation in health services Knowledge translation framework	None stated
Moore et al. (2017)^31^ QS: −	USA	Transitions of care	Carer, HCP	Transfer tool developed by authors (ADMIT ME)	Carers, nurses, first responders	Focus groups		None stated	None stated

*Note*: Local site liaison, part of implementation team in Luxford et al.,^29^ and Clinical Excellence Commission.^12^, ^27^ Quality score, taken from NICE (2012), graded ‘−’, ‘+’ or ‘++’.^17^

Abbreviations: HCP, healthcare professional; HP, healthcare passport; LSL, local site liaison; NHS, National Health Service; PLWD, person living with dementia; QS, quality score.

^a^ Research report.

^b^ Corresponding journal article.

^c^ Research report.

^d^ Corresponding journal article.

**Table 2:** Summary of multicomponent studies

Reference quality	Country	Setting	Perspective		Programme/document	Participants	Design	Theoretical models	Analysis
Ayton et al. (2019)^32^ QS: +	Australia	Hospital	Healthcare professionals/volunteers		Volunteer Dementia and Delirium Care (VDDC): − 1:1 companionship − Therapeutic activities − Personal profiles	Nurses, doctors, hospital volunteers in 3 focus groups; 7 interviews	Focus groups/interviews to explore future implementation of VDDC	Acceptability of Healthcare Interventions framework	Deductive coding using framework analysis
		Hospital	PLWD + carers			Interviews (*n* = 4)		Acceptability of Healthcare Interventions framework	Deductive coding using framework analysis
Ayton et al. (2019)^33^ QS: +	Australia	Hospital	Healthcare professionals/volunteers		Volunteer Dementia and Delirium Care (see above)	Nurses, doctors, hospital volunteers in 3 FGD (*n* = 9) and 8 interviews	Focus groups/interviews to explore future implementation of VDDC	Capability, Opportunity, Motivation‐Behaviour (COM‐B)	Deductive coding using framework analysis
		Hospital	PLWD + carers			Interviews (*n* = 4)		COM‐B	Deductive coding using framework analysis
Bolton et al. (2016a)^34^ QS: −	New Zealand	Hospice	Nurses		− Te Kete Marie: − This is me − Activity toolbox − Education − Interdisciplinary team member − Resource role − Environmental and reality orientation resources − Micromanagement of individual patients − Montreal Cognitive Assessment − Consumer feedback	Nurses, allied health professionals, doctors, volunteers, nonclinical staff (total *n* = 51 preintervention; *n* = 45 postintervention)	Plan Do Study Act cycle for toolkit development Evaluation survey online pre‐ and postimplementation	None stated	None stated
Bolton et al. (2016b)^35^ QS: +	New Zealand	Hospice	Carer		Te Kete Marie (see above)	10 Semi‐structured interviews for carers of people receiving TKM in the previous 12 months	Interviews following Te Kete Marie care in the previous 12 months	None stated	Thematic analysis
Edis et al. (2017)^36^ QS: −	England (NHS)	Hospital	Healthcare professionals		1. About Me 2. Dementia resource trolley 3. Online learning	Recovery room staff	Plan Do Study Act cycle of three initiatives; online survey before implementation; ‘feedback book’ for the trolley, not stated for other initiatives	None stated	None stated
Grealish et al. (2021^20^ QS: +	Australia	Hospital	PLWD + carers + healthcare professionals		This is me educational programme	PLWD + carer dyads (*n* = 18 dyads)	Personal profile audit Staff education training log Carer interviews HCP interviews Surveys postintervention	Implementation theory	Summaries of quantitative data Thematic content analysis
Parke et al. (2019)^37^ QS: +	Canada	Hospital	Carers		Hospital‐readiness tools 1. Be ready for an ED visit (orienting checklist) 2. My ready‐to‐go bag 3. About me 4. My medication 5. Who knows me best 6. My wishes 7. Plan ahead for going home	14 Carers in 2 focus groups	Interpretative qualitative exploratory design using focus groups, review of notes on tools, semi‐structured interview	None stated	Content analysis
Sampson et al. (2017)^38^ QS: +	England (NHS)	Hospital		Healthcare professionals, nonclinical hospital staff	Train the trainer model, including This is Me	Healthcare professionals; nonclinical hospital staff	Mixed methods Individual: Survey perceived sense of competence Ward: Person Interactions Environment Organization: use of This is Me tool; pre–postquestionnaires to local dementia lead System: Number of people completing training	Change framework	Thematic content analysis

*Note*: Quality score, taken from NICE (2012), graded ‘−’, ‘+’, or ‘++’^16^; Volunteer Dementia and Delirium Care from Ayton et al. papers.^32^

Abbreviations: COM‐B, Capability, Opportunity, Motivation—Behaviour; NHS, National Health Service; PLWD, person living with dementia; QS, quality score; VDDC, Volunteer Dementia and Delirium Care.

All studies were in Anglophone countries, with most set in the UK or Australia, and two papers from Canada reporting experiences from English‐ and French‐speaking communities. Experiences were included from PLWD, carers and HCPs. Most studies (single and multicomponent) evaluated their own PID or programmes; however, four studies using an existing PID utilized This Is Me, developed by the Alzheimer's Society.^22^, ^34^, ^35^, ^38^

The quality assessment of the included papers is provided in the first column of Tables 1 and 2. Of the 19 papers, none were assigned a quality assessment score of ++ (all or most of the checklist criteria have been fulfilled; where they have not been fulfilled, the conclusions are very unlikely to alter); 15 were assigned a quality assessment score of + (some of the checklist criteria have been fulfilled; where they have not been fulfilled, or not adequately described, the conclusions are unlikely to alter); and four were assigned an assessment score – (few or no checklist criteria have been fulfilled and the conclusions are likely or very likely to alter). Low‐quality studies were not included in the quotes in Table 3, and the authors have been mindful not to draw conclusions solely from them.

**Table 3:** Example quotes and barriers and facilitators to PID use, from PLWD, carers and HCPs, arranged by COM‐B domain

Perspective	Example barrier		Example facilitator
*Physical capability*			
PLWD	‘It will take initially some time to complete all the sections. This may not always be practical, particularly where people with dementia live on their own and may not be able to complete this task by themselves; or where carers are already overwhelmed. Also in cases, where family is unsupportive (“We are dependent on other people”)’. Leavey (2017)^10^		
Carer	‘As regards M., my husband, he won't be able to fill that in because he can't write now because he has problems with using his fingers and hands […]. Therefore, he wouldn't personally be doing this, it would be me’. Leavey, (2020)^28^		‘The carers also reported a need for staff to receive specific training and education on dementia and mental health’. Study authors quoting carers, Burton (2019)^25^
HCP	‘All hospital sites employ a high number of nursing staff in ED, therefore making it very difficult to educate all staff on the TOP 5 program. One site mentioned it is quite difficult getting on/staying on in‐service calendars to provide internal TOP 5 education to staff’. Study authors, quoting HCP, Clinical Excellence Commission (2015)^27^		‘The LSL noted that further TOP 5 education to the whole hospital would ensure the TOP 5 form is not thrown out and is kept with the patient as they move throughout the hospital to ensure continuity of care’. Study authors quoting HCP, Clinical Excellence Commission (2015)^27^
*Psychological capability*			
PLWD	‘[Participants] believed the healthcare professionals would already be sharing/recording the information without prompting’. Study authors quoting PLWD/carers, Leavey et al. (2020)^28^		‘It was pointed out that really all health and social care professionals involved in the care of the person would need to be familiar with the passport and its purpose, so that they know how they can/should contribute and know which sections of the passport pertain to them and need their input. Equally, so that they know where to find relevant and important information. Everyone involved should be informed and trained appropriately’. Study authors, quoting PLWD/carer, Leavey et al. (2017)^10^
Carer	‘I did find initially confusion in my mind. Because it starts off saying this is a form for use by the carer to help a person with dementia communicate with hospital staff. So I started to write it as carer and then at a particular point it starts to talk about the patient’. Burton et al. (2019)^25^		‘Family caregivers who had direct experience working in the healthcare sector tended to suggest that healthcare staff would find the passport very useful’.* S*tudy authors, about carers. Leavey et al. (2020)^28^
HCP	‘Not all wards in the participating hospital were aware of the TOP 5 program – therefore when a patient was transferred from ED/pre‐admission to another ward in the hospital with their TOP 5 form, it was sometimes ignored or thrown away by staff on these nonparticipating wards’. Study authors quoting HCP, Clinical Excellence Commission (2015)^27^		‘Findings… highlight the need to raise awareness of TOP 5 amongst paramedics… as they are a critical factor in the successful transfer of TOP 5 information between health care settings’. Study authors, about paramedics, Clinical Excellence Commission (2015)^27^
*Automatic motivation*			
PLWD	‘To put into it, and I think for families who are maybe struggling with the person with the diagnosis or a person who has just been recently diagnosed or is in…you know, in the middle of the illness, that this would maybe be something that wouldn't … that they wouldn't use’. Leavey et al. (2017)^10^	‘The most common response was “we will give it a go” – a tacit agreement to try it out’. Study authors, quoting PLWD and carers, Leavey et al. (2020)^28^	
Carer	‘This is what happened to us. Whenever [Name] was diagnosed we got bombarded with everything, which 90% of it was great but there was a couple that we couldn't just cope with, and that was one of them, you know, it was too much’. Leavey et al. (2017)^10^	‘The passport, at the moment, I think the passport will only be coming into usefulness now, because we are getting more people involved [] I can see that it would be useful it here's more going on, so you can keep track of it all’. Leavey et al. (2017)^10^	
HCP	‘At some participating hospitals, some staff members had the attitude of “not my job” or “not another form to complete” when conducting a TOP 5 for a patient. Therefore initiating TOP 5 was usually left for key staff’. Study authors, quoting HCP, Clinical Excellence Commission (2015)^27^	‘“I guess there is no reason why we couldn't actually complete it for them, if we find that we haven't got one in place already” They later discussed that it could be easier to complete the document in the community rather than in hospital, as the person was in their own environment and family may be present’. Baillie and Thomas (2020)^22^	
*Reflective motivation*			
PLWD	‘Nobody wanted to know’. Leavey et al. (2017)^10^	‘I think parts of that might be very therapeutic for somebody, a family member, to write down all the things that you want to [overtalking] That's the people that should be using that’. Leavey et al. (2017)^10^	
Carer	‘It's not so much reservations but will it actually make any difference to Mickey or myself, really? Will it actually make any difference? [] Well, I've only glanced at it but really I don't know’. Leavey et al. (2020)^28^	‘When she went into hospital, that whole explaining that she had dementia, there was nothing ever written down, you know? You were constantly explaining’. Leavey et al. (2017)^10^	
HCP	‘Another form! Will it improve the lives of patients and carers? The others rarely do!’. Leavey et al. (2020)^28^	‘I just think that people don't realise that these things are the psycho‐social aspect and people go, “we haven't got time to do it” but actually if you take those few seconds to fill it in, in the long term it will save time’. Baillie and Thomas (2020)^22^	
*Physical opportunity*			
PLWD	‘I think if you went into hospital, there's very few hospitals, in fairness, that's going to take the time to look even through that. No, they won't have time. Even though it's a brilliant idea’. Leavey et al. (2017)^10^		‘A HP on an electronic platform (updateable through GP computer systems or password protected access to essential information via NIECR or equivalent) would be better [ensuring legibility and confidentiality; not requiring patients to remember to bring their HP]. May not suit everyone, so could run have an optional paper version’. Leavey et al. (2017)^10^
Carer	‘I haven't really filled it out yet, I haven't had time, and I feel guilty about that. But I know that it's there and I often say “I must do that”.’ Leavey et al. (2017)^10^		‘Fairly self‐explanatory and [with] plenty of room for any information we may have had to put in’. Burton et al. (2019)^25^
HCP	‘…it was very helpful. But sometimes we didn't get time to fill it out’. Grealish et al. (2021)^20^		‘The nurse said she was able to complete a TOP 5 strategy form in less than 5 minutes, which assisted management of the patient in ED and during transfer to the surgical ward’. Study authors, quoting HCP, Clinical Excellence Commission (2015)^27^
*Social opportunity*			
PLWD	‘Some clients not comfortable with TOP 5 tag (which identified them as a TOP 5 client) visible in their home. Clients would become distressed if TOP 5 tag kept in a visible place inside their home (due to stigma), however would have been thrown it away if they found it hidden somewhere in their home’. Study authors, about PLWD, Clinical Excellence Commission (2015)^27^	‘People living with dementia need to know that [the PID] is widely used by all patients, so that they do not feel stigmatised by its use’. Study authors, about PLWD, Leavey et al. (2017)^10^	
Carer	‘All participants indicated the need for clarity regarding who is responsible for the tool document. Responsibility involved the following aspects: who fills out the document, who is the keeper of the information in the document for updates, and where the documents are located for timely access’. Study authors, about carers, Parke et al. (2019)^37^	‘They have it on the sheet… And they might not have time to look at it immediately. But now, they'll have to get used to working with this at some point in the emergency department’. Parke et al. (2019)^37^	
HCP	‘It's all very well us filling them out when they're here [in hospital], and then when they go home, how do we ensure that that then comes with them, because a lot of patients don't have that family network, it could get lost or is it the paramedic's responsibility for making sure they have it when they bring them here?’. Baillie and Thomas (2020)^22^	‘Key to the success of implementing TOP 5, was the integration of TOP 5 into established processes. For example, during admission and initial assessment processes, and as part of daily care’. Study authors, about HCP, Clinical Excellence Commission (2015)^27^	

Note: Data presented are taken from the analysis of single‐component studies only. Quotes from studies scoring ‘+’ or ‘++’ only. Healthcare passport, PID used in Leavey et al.^10^, ^28^ studies; local site liaison, part of implementation team in Luxford et al.^29^ and Clinical Excellence Commission^26^, ^27^; TOP 5 = PID used in Luxford et al.^29^; and Clinical Excellence Commission.^26^, ^27^

Abbreviations: COM‐B, Capability, Opportunity, Motivation—Behaviour; ED, emrgency department; HP, healthcare passport; LSL, local site liaison; PID, personal information document; PLWD, person living with dementia.

**Figure 2:**
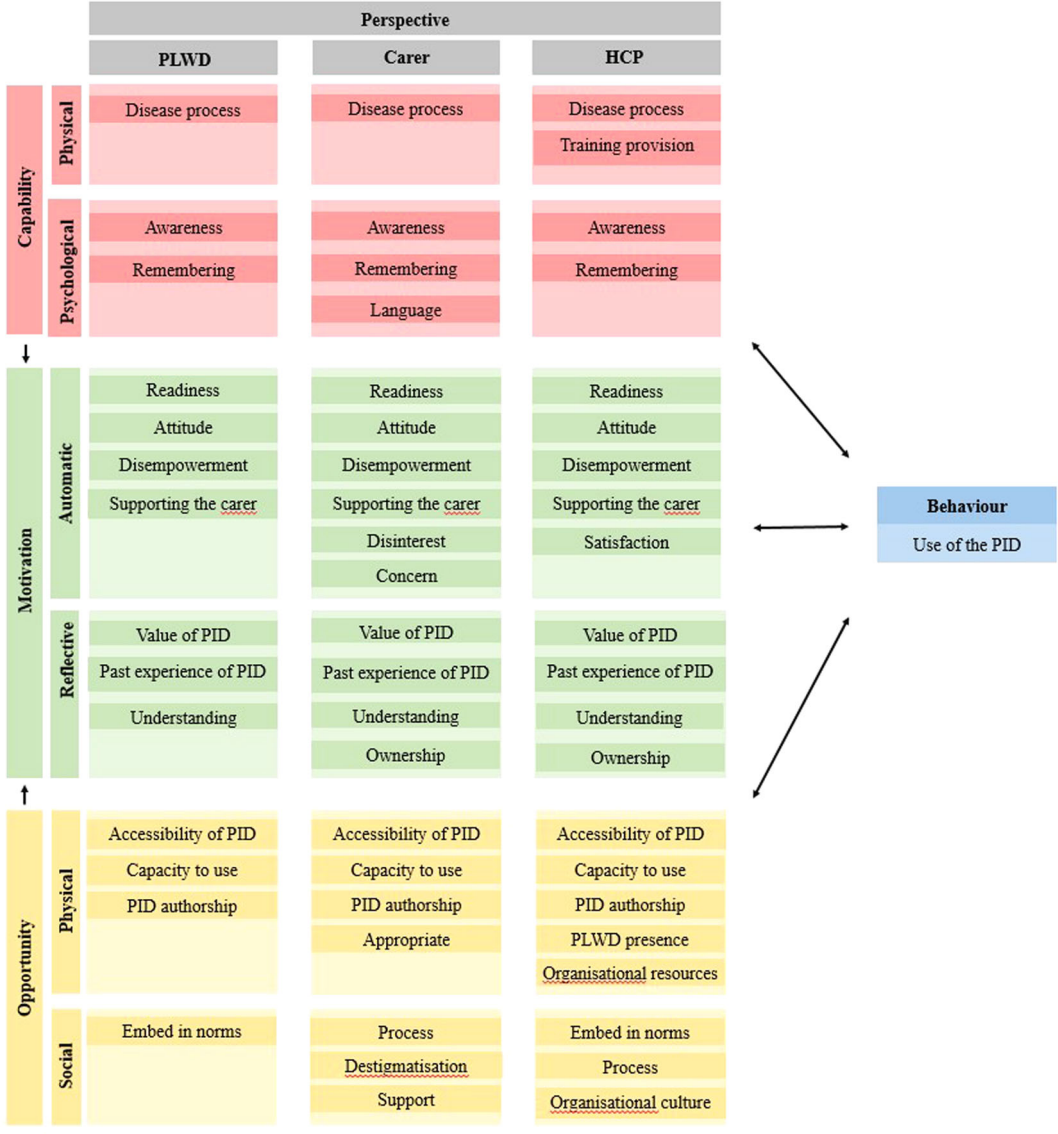
Themes from both single‐ and multicomponent studies mapped onto the COM‐B model of behaviour change. COM‐B, Capability Opportunity Motivation—Behaviour; HCP, healthcare professional; PID, personal information document; PLWD, people living with dementia

Figure 2 presents the factors for PID use, identified in the thematic analysis and mapped to the COM‐B model, presented by perspective (PLWD, carer, HCP). Table 3 provides example quotes, organized by COM‐B domain, illustrating the barriers and facilitators of PID use. For a more detailed table of themes and quotes, please see Table S3.

### 3.1 Physical capability

Attending training was predominantly discussed by HCPs, with difficulties attending training identified as a barrier,^27^ and embedding training into existing education seen as a facilitator.^27^ Completion of training was believed to enable a common understanding that would help HCPs keep the PID near the patient, and not throw the form away^27^—carers also commented on the need for a shared appreciation of the PID.^25^

The disease process was a barrier to PLWD using the PID, due to the physical and cognitive manifestations of dementia^10^, ^27^, ^28^ or comorbidities. PLWD may forget, or have difficulties writing.^10^, ^28^ These challenges were greater for PLWD who lived alone or those dependent on carers who were already overwhelmed.^10^

### 3.2 Psychological capability

HCPs identified that their own awareness of the PID is crucial,^10^, ^23^ for example, paramedics' awareness was seen to be necessary to transfer information between healthcare settings.^27^ HCPs who were aware would ensure that the PID is ‘kept with the patient’,^27^ and not ignored or thrown away.^27^ However, Leavey et al.^28^ described PLWD/carers with hesitant attitudes towards the PID, resistant to change despite increased awareness. HCPs, carers and PLWD had suggestions for increasing awareness, for example, using local team meetings,^27^ promotional material in patient‐facing areas^10^ or TV advertisements.^10^

Studies showed that remembering the PID is crucial to its use; for example, HCPs commented that PLWD need to take it with them.^10^ Suggestions to help HCPs, carers and PLWD to have the PID available included having it available on a mobile phone^10^; reminder stickers or posters in healthcare or care home settings^10^, ^27^; and alert systems on healthcare computers,^27^ and that carers could remind HCPs to use the PID.^23^, ^24^

Forgetfulness was a barrier as the PID was sometimes left at home,^10^, ^28^ not transferred between the care home and hospital^27^ or, once in the hospital, was forgotten and not used.^23^, ^27^ There was concern that PLWD would forget the existence of the PID and carers may be too stressed about the admission to remember it.^10^

### 3.3 Physical opportunity

Accessibility of the PID was a key enabler to PID use. The availability of blank forms in the community enabling PLWD and carers or care home staff to complete before hospital admission,^23^, ^24^ and completing the PID at the PLWD's home^10^, ^22^ were facilitators. Electronic access to blank PIDs (e.g., on a computer or a mobile app) was seen as helpful by PLWD, carers and HCPs;^10^, ^27^ however, some preferred paper formats.^10^ Some studies scanned completed paper PID into electronic systems, which was found to be helpful.^22^, ^23^, ^24^ Paper PIDs were found filed away in patient notes^22^; this was overcome by keeping them in a defined location.^27^, ^30^, ^31^

PLWD,^10^ carers^10^, ^25^, ^28^ and HCPs^10^, ^27^, ^28^, ^30^ all reported that lack of HCP time was a barrier to PID use. Some PIDs were more onerous to complete than others: shorter documents were generally preferred,^27^ whereas longer documents were seen as too cumbersome^10^, ^28^ and people were discouraged by the time required for PID completion.^10^ Furthermore, keeping the document up to date was seen as onerous.^10^ PLWD,^10^ carers^10^, ^25^ and HCPs^22^, ^27^ all reported competing priorities as a barrier, although the activities being prioritized varied. Policy changes such as the introduction of the 4‐hour rule in the emergency department where patients should be admitted or discharged within that time frame were considered to be barriers.^27^ PIDs that were easy to use were reported to have increased chance of use than those that were complex.^10^, ^22^, ^25^, ^27^, ^29^, ^30^, ^31^

The presence of carers was important: barriers to PID use included PLWD who were alone or lacked family,^22^, ^27^ and facilitators to its use were the presence of nominated individuals to take responsibility for the PID.^10^, ^22^, ^24^, ^27^, ^28^, ^31^ HCPs observed that they too were well placed to complete PIDs including in conjunction with carers.^22^, ^27^

Organizational‐level factors were noted to influence PID use, for example, prioritization of health service response to the Ebola crisis or health service reaccreditation requirements.^27^ Staff perceptions of small numbers of PLWD being admitted to hospital represented a barrier,^29^ but proactive identification of PLWD overcame this.^27^

### 3.4 Social opportunity

PIDs were seen as able to both cause^27^ and reduce stigma,^10^ and suggestions to reduce stigmatization included informing PLWD that the PID is commonly used.^10^ Embedding the PID into organizational norms and incorporating the PID into existing activities helped its use, for example, as patients were readmitted, bringing their existing PID with them.^24^ HCPs commented on a lack of process as a barrier, overcome by facilitators including keeping the PID with the PLWD throughout their journey,^24^, ^25^, ^27^ ensuring that the patient would be discharged with their PID^22^ and clarifying roles and responsibilities.^22^

A culture of collaboration enables PID use. HCPs stated that team working^27^, ^29^ and using existing communication networks including communication with carers^26^, ^27^, ^29^ were beneficial. Good leadership promoted PID use, through clinical champions, modelling desired actions, local implementation teams and senior management support.^27^, ^29^ Considering PIDs as a process of continuous improvement supported its development through regular review of the initiative,^27^ sharing of barriers^27^ and cultivating a supportive environment and culture.^10^, ^24^, ^30^

### 3.5 Automatic motivation

The desire and willingness of PLWD and carers to use the PID influence the acceptance of PIDs. The PID was seen as a reminder of illness by PLWD^10^ and an intrusion into normality by carers.^28^ For those with mild dementia, the PID was seen as a future necessity once dementia had progressed^10^, ^22^, ^27^, ^28^ and the PLWD/carer had come to terms with the diagnosis.^10^ Carer stress^10^, ^27^, ^28^ encompassed carer health and well‐being and the carer's capacity to cope. The strength of the carer–PLWD bond appears to be important in terms of PID use: where the bond was strong, PID use was better,^28^ but PIDs were used less where carers were overwhelmed or upset.^10^, ^27^

The attitudes of HCPs, carers and PLWD towards the PID were important. Some carers considered the PID a chore.^10^, ^28^ HCP buy‐in was important,^22^, ^29^ and some HCPs considered the PID as someone else's job.^27^ Concerns were raised about the PID: carers were worried about data protection,^28^ and HCPs feared for the legal status of PIDs.^28^ PLWD and carers were anxious or embarrassed to write in the PID.^10^, ^28^

### 3.6 Reflective motivation

The predominant theme within the synthesis was the value of the PID. PLWD, carers and HCPs had all imagined the positive impact that the PID could have on PLWD,^10^ on carers^10^ and on HCPs.^22^, ^25^, ^26^, ^30^, ^31^ For PLWD, the PID would improve patient‐centred care,^10^, ^22^, ^23^, ^25^, ^26^, ^27^, ^28^, ^29^, ^30^ help retain their identity^22^ could reduce behaviours that challenge^22^, ^25^, ^26^, ^27^, ^29^ and improve their comfort. Carers' stress could be reduced through their knowing that their loved one would receive improved quality of care,^27^ and that their loved one would be cared for in their absence.^10^, ^28^ HCPs found the document time‐saving,^22^, ^26^, ^30^ reduced workload,^31^ helped undertake the job^10^, ^22^, ^24^, ^27^, ^31^ and improved the quality of care that they were able to give.^22^, ^25^, ^26^, ^27^, ^29^, ^30^ HCP confidence improved with continued PID use.^27^, ^29^, ^30^ Communication between PLWD, carers and HCPs was improved,^26^, ^27^ for example, where the PLWD was unable to communicate for themselves.^10^, ^25^ There was an impact on the healthcare organization as well, with reductions in specialist nursing^27^ and complaints,^29^ and improved restructuring of the ward.^26^ Where barriers were identified, it was because the benefits of the PID had not been observed,^26^, ^27^, ^30^ scepticism that the PID would be used^28^ or that the benefit of the PID did not outweigh the burden of using it.^10^

Prior experience of similar initiatives influenced receptiveness to PID use, causing PLWD or carers to self‐censor,^28^ engage^27^ or disengage^10^, ^25^, ^28^ with PIDs.

Barriers were identified regarding the purpose,^23^, ^25^ use^25^, ^28^ and ownership^10^, ^28^ of the PID by HCPs, carers and PLWD. There was a belief amongst PLWD and carers that the PIDs were not needed, for example, that HCPs would already be sharing the information,^28^ or that the carers would be present for any hospital admissions for the PLWD.^27^ Carers may not have understood the purpose as they reported confusion around the perspective in which the PID should be completed.^25^ There was uncertainty over who had ownership of the PID^10^ and who would have access.^28^ Carers and HCPs provided suggestions on improvements to the PID to improve its relevance.^30^, ^31^

### 3.7 Multicomponent interventions

Themes from multicomponent analysis corroborated those identified from the single‐component studies; for example, in physical capability, the manifestations of dementia were identified as a barrier to PID use by carers^37^ and HCP training improved PID use.^38^ Carers described difficulties understanding the language of the PID.^37^ Clear ownership and responsibility was a facilitator.^34^, ^37^

Similar to single‐component interventions, accessibility of PID, for example, with electronic adaptation^34^ or a defined location^37^ represented enablers. Redundancy of the PID was noted in some contexts,^37^ with a suggestion to adapt the PID to the local environment. Lack of time was reported as a barrier.^36^, ^37^ HCPs reported a fear of losing a PID during transfers between wards and suggested inclusion with other documentation and clarity of responsibility.^36^ PIDs were perceived as normalizing dementia care.^37^ The documents would also enable carers to feel included in care.^37^

Carers were concerned about information required for the PID^32^ and whether the tool would be used.^37^ HCPs were perceived as disinterested by carers^37^; yet, HCPs reported feeling satisfied at being able to provide good care through using the PID.^34^ Concordant with single‐component intervention findings, the predominant theme was that of value of the PID with the potential to improve personalized care^33^, ^34^, ^35^, ^37^; support the PLWD's unique identity^34^, ^37^; and reduce or prevent responsive behaviours.^37^

## 4 DISCUSSION

This systematic review synthesized findings from 19 papers investigating barriers and facilitators to the use of PIDs for PLWD from the perspectives of PLWD, carers and HCPs. Understanding the component factors to the use of a PID is necessary to optimize their implementation, in line with recommendations from NICE^2^ and the Royal College of Psychiatrists,^9^ with the ultimate aim of improving the quality of person‐centred care for PWLD. PLWD are at higher risk of complications during hospitalization^2^, ^5^; PIDs aim to reduce preventable morbidity and mortality.

A wide range of findings were identified, ranging from practical (where to keep the PID), to conceptual and abstract factors (the PID symbolizing illness). Many barriers and facilitators identified were different experiences of the same phenomenon, for example, lack of time to use the PID versus the potential for the PID to be time‐saving.

A key finding was that PIDs were valued by PLWD, carers and HCPs. Experiences of PID use demonstrated benefits at multiple levels: PLWD received higher‐quality personalized care, carer stress was reduced and HCP could provide better quality of care. All perspectives commented on the improved communication and satisfaction with care. This echoed with the literature on PID use for other medical conditions.^39^

### 4.1 Relevance to practice

The COM‐B model is used for behaviour change intervention development.^12^ Some findings provide a direct opportunity to improve the use of PID, for example, ensuring that blank forms are available. Where nonmodifiable barriers have been identified by this review, such as the progressive nature of dementia, understanding interrelationships between wider barriers, facilitators and behaviours can aid intervention development to identify opportunities to mitigate some of the effects of a nonmodifiable barrier, for example, by considering the timing of the PID, supporting and empowering the carer or considering completion of the PID as a collaborative effort that included HCPs.

### 4.2 Role of the carer

The importance of supporting the carer was identified in this review: ‘the carer is the lynchpin’.^10^ Carers are required at key moments in PID use: authorship, ownership, bringing the PID to appointments and reminding HCPs. The PID is unlikely to be used where carers are overwhelmed or disengaged. However, carers have been called the ‘invisible second patients’, subject to significant strain, burnout and depression.^40^ Caring duties may prohibit working, with economic consequences.^41^ Thus, any intervention to improve PID use should have both PLWD and their carers at their core. Supporting carers is a key component of the NICE guidelines.^2^

### 4.3 Healthcare professionals

HCPs form one perspective in this review; however, different HCP cadres will have different interactions with the PLWD, carer and the PID, and observe different benefits and challenges. Healthcare assistants and nurses provide much of the ‘hands‐on’ essential care for inpatients and they should feel empowered to utilize the PID (e.g., through knowledge, access to the PID)^42^—but they do not feel empowered to bring about change.^43^ Other cadres of HCP are more influential in establishing organizational culture (e.g., those in positions of leadership and management) to enable and value nursing staff who spend time with PLWD to get to know them,^44^ for example, through PID.

HCPs wanted to attend PID training and stated that it would improve their confidence, corroborating research findings that HCPs are not adequately prepared to care for unwell PLWD,^43^ and the NICE guidelines advising specialized training on ‘understanding the person as an individual’.^2^ Specialized training in dementia care for HCPs is advised,^2^, ^45^ improves HCP attitudes^43^ and increases PID use.^38^ HCPs' attitudes were identified as a barrier to the use of patient‐held records in other studies—where the HCP did not understand the purpose of the document, or did not empower the patient as intended.^46^

### 4.4 Healthcare services

Hospitals are task orientated rather than person‐centred.^47^ PLWD, carers and HCPs all identified that PID can promote person‐centred care. Therefore, the organizational culture must support and value person‐centred care to promote quality of care.^47^ For example, studies have shown that getting to know PLWD on hospital wards was not prioritized by managers or ward staff, in part due to workload constraints.^44^ High HCP workload is a barrier to the use of other patient‐held records also.^46^ Yet, although time and workload constraints were identified by this review as barriers, others stated that the PID could be time saving. The healthcare service must provide adequate resources—including staff time and valuing the staff that provide it.^42^, ^45^ Reprioritization at an organizational level could facilitate better PID use.

Other research has advocated for senior support for personalized care.^39^, ^45^ Patient safety is inherent to any healthcare organization,^48^ and so this focus could be harnessed by the power of the PID to improve patient‐centred care.^45^ For example, Luxford et al. noted a reduction in chemical restraint since the introduction of their PID,^29^ an important outcome as medications are associated with increased morbidity and mortality.^49^

### 4.5 Overarching policy

A system‐wide approach is needed to embed the PID fully—PLWD receive care from a number of organizations, and PLWD may be transferred between settings. Expectations and understanding of the PID must align for all stakeholders for successful PID use. In accordance with our findings, reviews of other PIDs identified the importance of clarifying in advance the responsibility for the PID, its purpose and process, including embedding the PID within current work practices.^39^, ^46^

### 4.6 Review limitations

Difficulties were encountered during the literature search due to the lack of a unifying term for PID. There may be publication bias, for example, with internal service evaluations and audits of PID not having been published. Thus, some pertinent barriers and facilitators may not have been identified. However, a range of studies, heterogeneous in design, population and PID used, were identified. This review utilized an inclusive approach to garner the breadth of barriers and facilitators.

Four of the 19 studies were of low quality (scoring ‘−’ on quality appraisal), with the remaining 15 scoring ‘+’ and none scoring ‘++’.

There is no standard PID in use, and so the PID used differed between studies. Barriers and facilitators may depend on the PID used (e.g., some study participants complained that the PID was too long^28^; others stated that the PID was short and easy to use.^29^ This will limit comparability between studies—but also validates some findings as they are reproducible in different settings.

There is limited external validity of findings; they may need adaptation for consideration to context. For example, cultural differences between healthcare organizations or PLWD demographic groups may influence engagement with PID. Parke et al. had separate focus groups for English‐ and French‐speaking participants in Canada and noted that cultural differences mediated the appreciation of hospital‐readiness tools, including PID. Changes to the language used were advised by participants.^37^

The voice of PLWD remains quiet, especially in studies of PID within toolkits. Data were lacking on the influence of the stage of dementia on the barriers and facilitators to PID use. This may reflect difficulties in recruiting PLWD into research studies.

Finally, while mapping themes to the COM‐B model has benefits in terms of identifying future interventions to support PID use, some themes did not fit neatly within any one subcomponent of the model. In some ways, this reflects the nature of the COM‐B as the components influence each other (capability and opportunity can influence motivation). In our mapping, we have, for example, mapped remembering to use the PID to the psychological capability subcomponent, but it can also have resonance with physical opportunity if there are resources in the environment that might aid memory. The intersections across subcomponents reflect the limitations of mapping to a model in which components interact.

### 4.7 Implications for future research

Future research should consider the impact of the SARS‐CoV‐2 pandemic on PID use in dementia care, as visiting restrictions to homes and hospitals have changed, and so the relative value of the PID may have changed. The impact of PLWD and carer stress, HCP workload and organizational priorities may also have changed the use of PID in practice.

The impact of PID use on actual person‐centred care experienced by PLWD should be studied. Increased completion of the PID or increased availability of PID in hospital settings may not translate to actions undertaken by HCP (the ‘know‐do gap’).^50^

## 5 CONCLUSION

This systematic review has identified a broad range of barriers and facilitators to the use of PID. By mapping these onto the COM‐B framework, evidence‐informed initiatives can be developed to improve the use of PIDs in the care of PLWD.

## CONFLICTS OF INTEREST

The authors declare no conflicts of interest.

## Supporting information

Supporting information.

Supporting information.

Supporting information.

## Data Availability

All articles included in the review are referenced and publically available. An example search strategy is included in the Supporting Information Material.
