# Supplementary material for: Barriers and facilitators to the use of personal information documents in health and social care settings for people living with dementia: A thematic synthesis and mapping to the COM‐B framework
Source: Health Expect. 2022 Apr 12;25(4):1215–31. doi: 10.1111/hex.13497 (PMC9327869; doi:10.1111/hex.13497)
Supplement: Supplementary file 2 — Supporting information. [file HEX-25--s002.docx]

| Supplementary Material  Table 2: OVID Medline search strategy | | |
| --- | --- | --- |
| Searched 02.07.2020 | | |
| **Line** | **Search Term** | **Results** |
| 1 | Passport*.ti,ab | 945 |
| 2 | ((personal information) adj1 (sheet* or document* or profile*)).ti,ab | 7 |
| 3 | (profile* adj2 (healthcare or health or care or dementia)).ti,ab | 5969 |
| 4 | (“read about me” or snowflake or “getting to know” or “this is me” or “what I need you to know” or “butterfly scheme” or “forget me” or “reach out to me” or “see who I am” or “my care matters” or mycarematters or “Top 5”).ti,ab | 48673 |
| 5 | Communication/ | 83642 |
| 6 | (Communication adj2 (tool* or doctor or patient)).ti,ab | 10229 |
| 7 | (tool* adj1 clinical).ti,ab | 6937 |
| 8 | “continuity of patient care”/ | 19045 |
| 9 | Patient Transfer/ | 8400 |
| 10 | (transfer agreement*).ti,ab | 140 |
| 11 | (care adj2 transition).ti,ab | 1843 |
| 12 | (record* adj2 (patient or family)).ti,ab | 19278 |
| 13 | patient-centered care/ | 19309 |
| 14 | Personhood/ | 3970 |
| 15 | OR/1-14 | 217424 |
| 16 | dementia/ or nglish disease/ | 135732 |
| 17 | (dementia or (cognit* adj1 impair*) or (memory adj1 impair*)).ti,ab | 168650 |
| 18 | OR/16-17 | 237765 |
| 19 | “quality of health care”/ or program evaluation/ or quality improvement/ or Quality Indicators, Health Care/ | 166423 |
| 20 | (barrier* or nglish* or Effectiv* or Evaluat* or Implement* or Experience* or perception*).ti,ab | 6760590 |
| 21 | “evaluation studies”.pt. or evaluation studies as topic/ or Pilot projects/ or program evaluation/ or Intervention Studies/ | 300926 |
| 22 | OR/19-21 | 6958287 |
| 23 | 15 AND 18 AND 22 | 1685 |
| 24 | limit 23 to (English language ) | 1549 |
| 25 | limit 24 to yr=”2010 -Current” | 1108 |
