# Supplementary material for: Barriers and facilitators to the use of personal information documents in health and social care settings for people living with dementia: A thematic synthesis and mapping to the COM‐B framework
Source: Health Expect. 2022 Apr 12;25(4):1215–31. doi: 10.1111/hex.13497 (PMC9327869; doi:10.1111/hex.13497)
Supplement: Supplementary file 3 — Supporting information. [file HEX-25--s001.docx]

| Supplementary Material Table 3. Illustrative quotes for barriers and facilitators to PID use, from PLWD, carers, and HCPs, arranged by theme within each COM-B domain | | | |  |
| --- | --- | --- | --- | --- |
| **Theme** | **Perspective** | **Example Barrier** | **Example Facilitator** |  |
| **Physical Capability** | | | | |
| Provision of training for HCP | Carer |  | “*The carers also reported a need for staff to receive specific training and education on dementia and mental health”* - study authors, quoting carers, Burton *et al*., 2019^26^ |  |
|  | HCP | “*All hospital sites employ a high number of nursing staff in ED, therefore making it very difficult to educate all staff on the TOP 5 program. One site mentioned it is quite difficult getting on / staying on in-service calendars to provide internal TOP 5 education to staff*” - study authors, reporting HCP, Clinical Excellence Commission, 2015^25^ | “*The LSL noted that further TOP 5 education to the whole hospital would ensure the TOP 5 form is not thrown out and is kept with the patient as they move throughout the hospital to ensure continuity of care*” - study authors quoting HCP, Clinical Excellence Commission, 2015^25^ |  |
| Disease process prohibit PLWD using PID | PLWD | *“It will take initially some time to complete all the sections. This may not always be practical, particularly where people with dementia live on their own and may not be able to complete this task by themselves; or where carers are already overwhelmed. Also in cases, where family is unsupportive (“We are dependent on other people.”).” –* PLWD, Leavey *et al.,* 2017^10^ |  |  |
|  | Carers | *“As regards M., my husband, he won’t be able to fill that in because he can’t write now because he has problems with using his fingers and hands […]. Therefore, he wouldn’t personally be doing this, it would be me”* - carer in community, Leavey *et al.*, 2020^27^ |  |  |
|  | HCP | *“Staff noted that it could be difficult when developing the strategies with the carer if the patient is present and did not have an insight into their condition or dementia diagnosis”* - HCP in hospital, Clinical Excellence Commission, 2015^32^ |  |  |
| **Psychological Capability** | | | | |
| Awareness of PID | PLWD / carers | *“In each of these cases, the opinion of those individuals did not change with further explanation of the passport*” – study authors about PLWD/carers in community, Leavey *et al*., 2020^27^ | *“Carers are also becoming more aware of the passport and now remind staff to look at it”* – study authors, about carers, Brooker *et al*., 2014^29^ |  |
|  | HCP | “*Not all wards in the participating hospital were aware of the TOP 5 program – therefore when a patient was transferred from ED / pre-admission to another ward in the hospital with their TOP 5 form, it was sometimes ignored or thrown away by staff on these nonparticipating wards”* - study authors, about HCP in hospital, Clinical Excellence Commission, 2015^25^ | “*Findings…highlight the need to raise awareness of TOP 5 amongst paramedics…as they are a critical factor in the successful transfer of TOP 5 information between health care settings”* - study authors, about paramedics involved in patient transfer, Clinical Excellence Commission, 2015^25^ |  |
| Remembering the PID | PLWD/carer | “*Over the study period, many people stated simply that they forgot to use the passport which was often put away “for safekeeping*”” - study authors, about PLWD and carers in community, Leavey *et al.,* 2020^27^ | *“A HP on an electronic platform … would be better [ensuring legibility and confidentiality; not requiring patients to remember to bring their HP].”* - PLWD in community, Leavey *et al.,* 2017^10^ |  |
|  | Carer | “*My fear at the outset, with Dad being a dementia patient, is that he will forget to take it with him*”- carer in community, Leavey *et al*., 2017^10^ | *“It also helped to make carers more aware of the passport and has resulted in them reminding staff about the passport, especially when they think it would be beneficial to their relative's care”* – carers in hospital, Bray *et al.,* 2015^28^ |  |
|  | HCP | “*The HP only works if patients remember to bring it in”* - study authors, quoting GPs in primary care, Leavey *et al.,* 2017^10^ | “*Due to this service linkage, staff at both hospital and community sites were able to advocate for permission to incorporate a TOP 5 alert in the EMR, which would benefit both Hospital G and Community D*” - study authors, about HCP, Clinical Excellence Commission, 2015^25^ |  |
| **Physical Opportunity** | | | | |
| Presence of PLWD | HCP | *“At one site staff mentioned that unfortunately some staff members do not check the patient’s history properly, therefore the flagging of potential TOP 5 patients is sometimes missed”* – study authors about HCP in hospital, Clinical Excellence Commission, 2015^25^ | *“Hospital sites with ASET staff who were willing to be involved in the TOP 5 process found more potential patients were identified and captured for TOP 5, as ASET routinely provide specialised care to older persons presenting to the ED”* – study authors about HCP in hospital, Clinical Excellence Commission, 2015^25^ |  |
| Accessibility of PID | PLWD | “*I'd prefer to write on paper. I'm not advanced enough on this laptop so I just prefer to write on paper.”* - PLWD in community, Leavey *et al.,* 2017^10^ | “*A HP on an electronic platform (updateable through GP computer systems or password protected access to essential information via NIECR or equivalent) would be better [ensuring legibility and confidentiality; not requiring patients to remember to bring their HP]. May not suit everyone, so could run have an optional paper version*” - PLWD in community, Leavey *et* al., 2017^10^ |  |
|  | Carer |  | “*If the app was along the lines of that medical thing or Facebook where you could put in your name and National Insurance Number and bring up your passport*” – Carer, Leavey  *et al.,* 2017^10^ |  |
|  | HCP | *“Staff also identified the importance of a completed “This is me” not being filed away in case notes where it could not be accessed”* – study authors quoting HCP, Baillie & Thomas, 2020^21^ | *“‘This is me could be scanned into the electronic patient record (EPR). They further discussed whether it could be completed and uploaded to the EPR while the patient is in the community, as a paper version could be difficult to find and: ‘if somebody is acutely unwell you’re not going to faff around for a bit of paper’*” - study authors, about HCP, Baillie & Thomas, 2020^21^ |  |
| Capacity to use | PLWD | *“I think if you went into hospital, there’s very few hospitals, in fairness, that’s going to take the time to look even through that. No, they won’t have time. Even though it’s a brilliant idea.”* – PLWD in community, Leavey *et al.,* 2017^10^ | “*I think you know a smaller, condensed version of what’s there…With the really important information that needs to be shared amongst GPs and healthcare people”* - PLWD, Leavey *et al.,* 2017^10^ |  |
|  | Carer | “*I haven't really filled it out yet, I haven't had time, and I feel guilty about that. But I know that it's there and I often say ‘I must do that’*.” – carer in community, Leavey *et al.,* 2017^10^ | *“If it was done easier, because all that c..p there that they have in it needs to be shorter and more…Then it might work”* – carer, Leavey *et al.,* 2017^10^ |  |
|  | HCP | “*ED staff are already quite busy with clinical requirements and do not always have time to sit down with the carer and develop TOP 5 strategies. The introduction of the 4-hour rule in ED in public hospitals means patients have to be either admitted or discharged from ED within 4-hours reducing the amount of time staff have to achieve their clinical tasks for a patient”* – study authors reporting HCP in hospital, Clinical Excellence Commission, 2015^25^ | “*The nurse said she was able to complete a TOP 5 strategy form in less than 5 minutes, which assisted management of the patient in ED and during transfer to the surgical ward”* - study authors, about HCP, Clinical Excellence Commission, 2015^25^ |  |
| Authorship of the PID | PLWD | “*There were a high number of clients with dementia who lived alone without a carer”* – study authors about PLWD, Clinical Excellence Commission, 2015^25^ |  |  |
|  | Carer | “*Some clients, did have carers however often their carers also had a mental illness*” – study authors, Clinical Excellence Commission, 2015^25^ | “*As regards M., my husband, he won’t be able to fill that in because he can’t write now because he has problems with using his fingers and hands […]. Therefore, he wouldn’t personally be doing this, it would be me*” - carer in community, Leavey *et al.,* 2020^27^ |  |
|  | HCP | *“Staff discussed that completing the document was problematic when there was no family*” – study authors, about HCP in hospital, Baillie & Thomas, 2020^21^ | “*Therapists discussed that they could involve other people in completing the documents, for example, a neighbour might know what TV programmes the person likes and what calms them down*” - study authors, about HCP in hospital, Baillie & Thomas, 2020^21^ |  |
| Organisational resources | HCP |  | “*During the TOP 5 study period other issues arose that took priority over TOP 5 for staff e.g. Ebola crisis, hospital re-accreditation etc*” – study authors, Clinical Excellence Commission, 2015^25^ |  |
| **Social Opportunity** | | | | |
| Embed in norms | PLWD | “*Some clients not comfortable with TOP 5 tag (which identified them as a TOP 5 client) visible in their home. Clients would become distressed if TOP 5 tag kept in a visible place inside their home (due to stigma), however would have been thrown it away if they found it hidden somewhere in their home”* - study authors, about PLWD in community, Clinical Excellence Commission, 2015^25^ | “*People living with dementia need to know that [the HP] is widely used by all patients, so that they do not feel stigmatised by its use”* - study authors, about PLWD, Leavey *et al.,* 2017^10^ |  |
|  | HCP | *“It’s all very well us filling them out when they’re here [in hospital], and then when they go home, how do we ensure that that then comes with them, because a lot of patients don’t have that family network, it could get lost or is it the paramedic’s responsibility for making sure they have it when they bring them here?”* - HCP in hospital, Baillie & Thomas, 2020^21^ | “*Key to the success of implementing TOP 5, was the integration of TOP 5 into established processes. For example, during admission and initial assessment processes, and as part of daily care”* – study authors about HCP in aged care facilities, Clinical Excellence Commission, 2015^25^ |  |
| Organisational culture | HCP |  | *“Staff were encouraged to share their barriers with the CEC project team and other participating sites, to work through possible solutions, to find out how other sites had overcome similar barriers, and to share learnings. Most sites were able to overcome their identified barriers in some way or develop a solution that worked with their own internal process.”* – study authors about HCP, Clinical Excellence Commission, 2015^25^ |  |
| **Automatic Motivation** | | | | |
| Readiness | PLWD | “*To put into it, and I think for families who are maybe struggling with the person with the diagnosis or a person who has just been recently diagnosed or is in…you know, in the middle of the illness, that this would maybe be something that wouldn’t … that they wouldn’t use”* – PLWD, Leavey *et al.,* 2017^10^ |  |  |
|  | Carer | “*We also noted that some couples fearing the loss of their “old” relationship strove to maintain a “normality” which, in some instances, meant minimising external intrusion. With implications for the healthcare passport and for care generally perhaps, several couples suggested that they try and manage things themselves. “We handle things ourselves. As far as we can, we’ll do it. Wherever she goes, I go now””* - study authors, followed by a quote from a carer in the community, Leavey *et al.,* 2020^27^ | “*The passport, at the moment, I think the passport will only be coming into usefulness now, because we are getting more people involved [ ] I can see that it would be useful it here’s more going on, so you can keep track of it all*” – carer, Leavey *et al.,* 2017^10^ |  |
|  | HCP | *“- We had a lady, she’s got dementia, obvious it’s in beginning stage, and I said why has she not got a “This is me” document? It was like: “she doesn’t really need it”. I said but what if later she gets readmitted, her dementia is worse, you’ve got some reference point.*  *- They can be updated as well on new admissions.*  *- The moment a person is diagnosed they should have it.”* - HCPs in hospital, Baillie Thomas, 2020^21^ |  |  |
| Attitude | Carer | ““*Why do you think you might not have wanted to use it?” “To be honest about it, I'd be more lazy than anything else*.” - carer in community, Leavey *et al.,* 2017^10^ | “*The most common response was “we will give it a go” – a tacit agreement to try it out*” - study authors, about PLWD and carers in community, Leavey *et al.,*  2020^27^ |  |
|  | HCP | “*At some participating hospitals, some staff members had the attitude of “not my job” or “not another form to complete” when conducting a TOP 5 for a patient. Therefore initiating TOP 5 was usually left for key staff”* - study authors, about HCP in hospital, Clinical Excellence Commission, 2015^25^ | “”*I guess there is no reason why we couldn’t actually complete it for them, if we find that we haven’t got one in place already” They later discussed that it could be easier to complete the document in the community rather than in hospital, as the person was in their own environment and family may be present”* - community HCP, Baillie & Thomas, 2020^21^ |  |
| Disempowerment | PLWD | “*I can’t spell properly, that’s why I don’t write*” - PLWD in community, Leavey *et al.,* 2017^10^ |  |  |
|  | Carer | “*No, no, and you know, we don’t like to have a document like this lying about the house so that other people, for whom it is not their business, may come across it and see what’s going on or what [PLWD]’s drug regime is”* - carer in community, Leavey *et al.,* 2020^27^ |  |  |
|  | HCP | “*GPs…were concerned about its legal status in the event of medical complications or patient complaints”* - study authors, about GPs in the community, Leavey *et al*., 2020^27^ |  |  |
| Supporting the carer | PLWD/carer | “*A weak patient-carer dyad: Where the carer is in poor health, mentally and/or physically; is (di-) stressed, and/or overwhelmed, and does not cope well, supportive capacity is lacking. This may be accompanied by a weak relational bond.*” - study authors, Leavey *et al.,* 2017^10^ | “*We noted warmth between dyads in most of the interviews and this bond appears to assist in using the passport*” – study authors, Leavey *et al.,* 2020^27^ |  |
|  | Carer | “*This is what happened to us. Whenever [Name] was diagnosed we got bombarded with everything, which 90% of it was great but there was a couple that we couldn’t just cope with, and that was one of them, you know, it was too much*” – carer in community, Leavey *et al.,* 2017^10^ |  |  |
|  | HCP | “*Sites noted that the carer is often upset/overwhelmed in an ED environment which therefore made it hard to develop strategies with them*” – HCP in hospital, Clinical Excellence Commission, 2015^25^ |  |  |
| **Reflective Motivation** | | | | |
| The value of PID | PLWD |  | “*I think parts of that might be very therapeutic for somebody, a family member, to write down all the things that you want to [overtalking] That’s the people that should be using that.”* – PLWD in community, Leavey *et al.,* 2017^10^ |  |
|  | Carer | “*It’s not so much reservations but will it actually make any difference to Mickey or myself, really? Will it actually make any difference? [ ] Well, I’ve only glanced at it but really I don’t know”* - carer in community, Leavey *et al*., 2020^27^ | “*When she went into hospital, that whole explaining that she had dementia, there was nothing ever written down, you know? You were constantly explaining*.” - carer, Leavey *et al.,* 2017^10^ |  |
|  | HCP | “Mostly, however, most expressed doubt about the additional bureaucracy. Another form! Will it improve the lives of patients and carers? The others rarely do!” - GP in primary care, Leavey *et al*., 2020^27^ | “*I just think that people don’t realise that these things are the psycho-social aspect and people go, “we haven’t got time to do it” but actually if you take those few seconds to fill it in, in the long term it will save time”* - HCP in hospital, Baillie & Thomas, 2020^21^ |  |
| Understanding the PID | PLWD/carer | *“[Participants] believed the healthcare professionals would already be sharing/recording the information without prompting*” - study authors, about PLWD/carers in community, Leavey *et al.,* 2020^27^ |  |  |
|  | Carer | “*I did find initially confusion in my mind. Because it starts off saying this is a form for use by the carer to help a person with dementia communicate with hospital staff. So I started to write it as carer and then at a particular point it starts to talk about the patient*” - carer in community, Burton *et al.,* 2019^26^ | *“Family caregivers who had direct experience working in the healthcare sector tended to suggest that healthcare staff would find the passport very useful” –* study authors, about carers, Leavey *et al*., 2020^27^ |  |
|  | HCP |  | *“Care providers indicated that the plan was a helpful tool because […] the different sections were relevant” –* study authors, about HCP in care homes, McGilton *et al.,* 2017^31^ |  |
| Past experience | PLWD | “*Nobody wanted to know*” - PLWD in community, Leavey *et al*., 2017^10^ |  |  |
|  | Carer | “*They did try, however, taking the passport to various health appointments but reported that no-one was prepared to engage with it. They returned the HP without entries. “Don’t talk about the passport – do not talk about the frigging passport!””* - study authors, with a quote from a carer in the community, Leavey *et al.,* 2017^10^ |  |  |
|  | HCP | “*The idea sounds great in theory but my experience of patient held booklets is that they are rarely ever used except in the context of the first years of life when usually mothers bring them to child health appointments for vaccinations but never bring them to other appointments. Maternity booklets are similarly only brought to these appointments which expectant mothers thing are directly related to antenatal care but not to other appointments*” - GP in Primary Care, Leavey *et al.,* 2017^10^ | “*Staff members who found the process easy to implement due to other projects implemented at the RACF eg personalised charts, “who am I? programs”, etc”* - study authors, about HCP in residential aged care facilities, Clinical Excellence Commission, 2015^25^ |  |
| **Notes.** CEC = Clinical Excellence Commission; HCP = healthcare professional; HP = healthcare passport, PID used in Leavey *et al.* studies;^11,27^ LSL = local site liaison, part of implementation team in Luxford *et al*.,^32^ and Clinical Excellence Commission;^12,25^ PLWD = person living with dementia; TOP 5 = PID used in Luxford *et al*.,^32^ and Clinical Excellence Commission^12,25^ | | | |  |
